# Supplementary material for: A Na,K-ATPase–Fodrin–Actin Membrane Cytoskeleton Complex is Required for Endothelial Fenestra Biogenesis
Source: Cells. 2020 Jun 3;9(6):1387. doi: 10.3390/cells9061387 (PMC7349347; doi:10.3390/cells9061387)
Supplement: Supplementary file 1 [file cells-09-01387-s001.zip › cells-810918_Supplementary material/cells-810918_Supplementary material (Fig and Tab).docx]

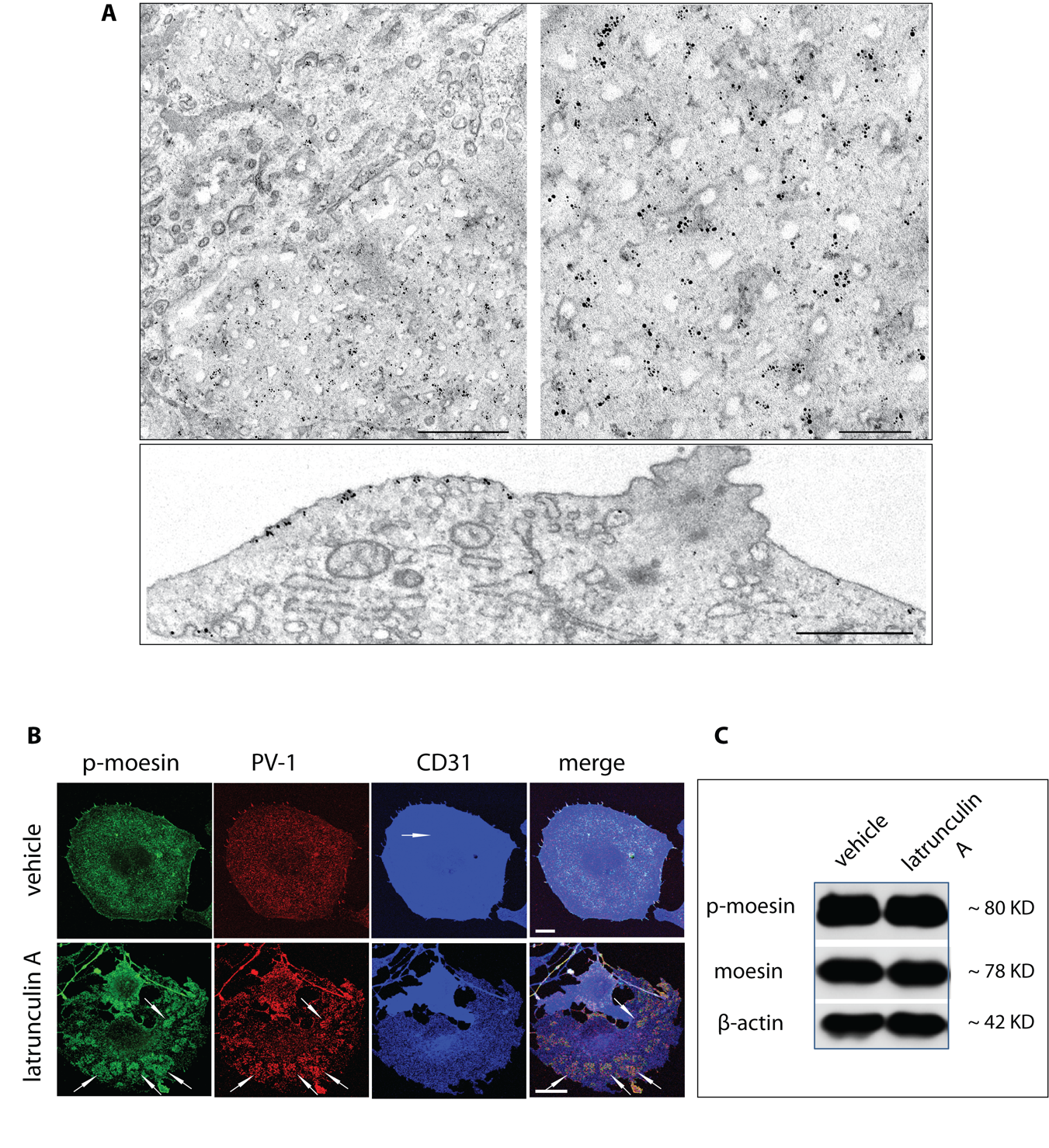


**Figure S1.** (related to Figure 2). Phosphorylated moesin is located in the sieve plate proximal to the fenestral pore. A, pre-embedding immunolabeling EM reveals that moesin is enriched in the fenestral sieve plate (denoted by dashes in top-left panel; scale bar, 200 nm); moesin was not present on the fenestral pore, but rather in electron dense patches adjacent (arrows in top-right panel; scale bar, 200 nm). Outside the sieve plate, moesin labelling was primarily localized to the plasma membrane (arrows in bottom panel; scale bar, 500 nm). B, a phospho-specific antibody reveals that phosphorylated moesin (p-moesin) redistributed into PV-1-positive fenestral sieve plates in LtA-treated bEND5 cells (arrows, sieve plates; scale bar, 10 μm). C, western blotting for phosphorylated moesin showed no change in the overall levels of p-moesin between non-induced and induced bEND5 cells.

**Table S1.** Proteins screened for cellular distribution in fenestrated bEND5 cells (Related to Figure 2).

| **Function** | **Candidates from proteomic analysis and literature search** |
| --- | --- |
| Cytoskeletal association | coffilin; myosin light chain IIA and IIB; moesin; radixin; ezrin; merlin; annexin II; twifilin; filamin-1; transgelin; cortactin; vimentin; desmin; VE-Cadherin; arps; integrins; EHM2 |
| Membrane remodelling  / endocytosis | moesin; radixin; ezrin; annexin II; caveolin; CD44; CD31; paralemmin; neocleotide diphosphate kinase (nm-23); EHM2; coat proteins II (COPII): sec23; sec31A and SAR1; ERGIC-53 (ER-Golgi intermediate compartment 53); lamp1; EEA1 |
| Membrane fusion | Rab 4/5; exocyst components; syntaxins 1.4 and 5A |
| Cell spreading | FAK (focal adhesion-associated kinase); tanlin; vinculin |
| High membrane curvature generation / maintenance | ER (endoplastic reticulum) proteins: calreticulin, calnexin and KDEL; reticulon 4 and DP-1; reticulon 2 and 3; atlastin; IRS p58/p53 |
| Transcription / translation | Putative RNA-binding protein 3; hnRNP-K (heterogenous nuclear ribonucleoprotein K) |
| Metabolism | A-enolase |
| Others | NPC (nuclear pore complex) proteins; aquaporin 1 and 4; etc. |
